# Cloning and Characterization of Low-Molecular-Weight Glutenin Subunit Alleles from Chinese Wheat Landraces (*Triticum aestivum* L.)

**DOI:** 10.1155/2014/371045

**Published:** 2014-04-10

**Authors:** Hongqi Si, Manli Zhao, Xin Zhang, Guoliang Yao, Genlou Sun, Chuanxi Ma

**Affiliations:** ^1^School of Agronomy, Anhui Agricultural University, Hefei 230036, China; ^2^Key Laboratory of Wheat Biology and Genetic Improvement on South Yellow & Huai River Valley, Ministry of Agriculture, Hefei 230036, China; ^3^Biology Department, Saint Mary's University, Halifax, NS, Canada B3H 3C3

## Abstract

Low-molecular-weight glutenin subunits (LMW-GS) are of great importance in processing quality and participate in the formation of polymers in wheat. In this study, eight new LMW-GS alleles were isolated from Chinese wheat landraces (*Triticum aestivum* L.) and designated as *Glu-A3-1a*, *Glu-A3-1b*, *Glu-B3-1a*, *Glu-B3-1b*, *Glu-B3-1c*, *Glu-D3-1a*, *Glu-D3-1b*, and *Glu-D3-1c*, which were located at the *Glu-A3*, *Glu-B3*, and *Glu-D3* loci, respectively. Based on the proteins encoded, the number of deduced amino acids of *Glu-B3* alleles was approximately 50 more than those of *Glu-A3* and *Glu-D3* alleles. The first cysteine of *Glu-A3* and *Glu-D3* alleles was located at the N-terminal domain, while that of *Glu-B3* alleles was found in the repetitive domain, which may lead to the different functioning in forming disulfide bonds. All the eight genes were LMW-m types and the new allele of *Glu-B3-1a* which had nine cysteine residues may be the desirable LMW-GS gene for improving bread-making quality.

## 1. Introduction

Wheat dough possesses unique viscoelastic properties determined by the structure and interaction of storage proteins in making bread and noodles [1]. Wheat storage proteins are mainly composed of monomeric gliadins and polymeric glutenins [2, 3], and these glutenins are divided into the low-molecular-weight glutenin subunits (LMW-GS) and the high-molecular-weight glutenin subunits (HMW-GS) according to their electrophoretic mobility in sodium dodecyl sulfate-polyacrylamide gel electrophoresis (SDS-PAGE) [4]. The HMW-GS have been recognized as the major determinants of dough and gluten properties [5], but LMW-GS also play an important role in determining wheat dough's viscoelastic properties [6].

The LMW-GS genes are encoded by* Glu-A3*,* Glu-B3*, and* Glu-D3* loci on the short arms of chromosomes 1A, 1B, and 1D, respectively, in hexaploid wheat [7]. Allelic variation in LMW-GS is generally accepted to have an important effect on wheat processing quality [8].* Glu-A3a*,* Glu-B3d*, and* Glu-D3a* have a better effect on dough strength than other alleles at the* Glu-3* loci. The effects of* Glu-A3d*,* Glu-B3i*,* Glu-A3d*, and* Glu-B3d* contribute most to dough extensibility and Zeleny sedimentation volume, while* Glu-D3* loci do not have a significant effect on either [9]. According to He et al. [10], noodle quality is decided by the protein subunits of* Glu-A3d* and* Glu-B3d*, and* Glu-A3d* is significantly more important in this process than other alleles. Si et al. [11] reported that* Glu-B3b*,* Glu-B3g*, and* Glu-B3h* significantly heightened the SDS sedimentation volume, while* Glu-B3a*,* Glu-B3c*, and* Glu-B3j* significantly lowered the SDS sedimentation volume. So far, no consistent conclusion has been reached regarding the influence of LMW-GS allelic variation on wheat processing quality, perhaps due to the different materials used, and the interaction between genotypes and the environment.

Although the mobility of many LMW-GS subunits is very similar and sometimes overlapped with each other in SDS-PAGE, a lot of researches have recently been conducted on LMW-GS genes by allelic-specific polymerase chain reaction (PCR) in common wheat and related species [12–14], which lead to a better understanding of the function, structure, and diversity of LMW-GS.

The landraces possess many useful traits that have been lost in modern cultivars. The phenological, morphological, physiological, and quality traits in landraces are genetically diverse [15]. Although more than 15 LMW-GS genes have been found so far in wheat [16], further studies of the diversity of these LMW-GS genes and cloning of new and rare alleles are interesting and challenging. In this study, we isolated and characterized eight new alleles located at* Glu-A3*,* Glu-B3*, and* Glu-D3* loci from landraces for which no LMW-GS genes have been previously detected by the corresponding molecular markers [17–19].

## 2. Materials and Methods

### 2.1. Plant Materials

The landraces Jiangdongmen, Daqingmang, Hongjinbaoyin, Dabaimai, Hongmangzi, Hongdougou, and Baimangmai used in this study were kindly provided by the National Gene Bank of China, Institute of Crop Science, CAAS, China. In our previous research, none of the LMW-GS genes were detected in the varieties Daqingmang and Hongjinbaoyin using the PCR markers for* Glu-A3* [17] alleles, the* Glu-B3* [18] alleles from the varieties Hongdougou, Dabaimai, and Hongmangzi, or the* Glu-D3* [19] alleles from the varieties Jiangdongmen, Daqingmang, and Baimangmai. To determine whether the genes at the* Glu-A3*,* Glu-B3*, and* Glu-D3* loci corresponding to the above varieties are missing or whether it is a novel gene, we used locus-specific primers to amplify the LMW-GS genes specifically for the* Glu-A3*,* Glu-B3*, and* Glu-D3* loci.

### 2.2. Genomic DNA Extraction and PCR Amplification

Genomic DNA was isolated from single dry seeds according to the procedure of SDS-phenol-chloroform with minor modification. Six locus-specific primer sets, Glua3f1/Glua3r2 (R: GTACGCTTTTGTAGCTTGTGC, F: GATGCCAACGCCTAATGGCACAC) [17], 5/7 (R: TCCTGAGAAGTGCATGACATG, F: GTAGGCACCAACTCCGGTGC) [20], and 3/4 (R: TTGTAGAAACTGCCATCCTT, F: GTCACCGCTGCATCGACATA) [20], were used for amplifying LMW-GS genes at the* Glu-A3* locus on chromosome 1A and the* Glu-B3* and* Glu-D3* loci on chromosomes 1B and 1D, respectively. The locus-specific primers were synthesized by Shanghai Sangon Biological Engineering & Technology and Service Ltd. (http://www.sangon.com/). PCR amplification was performed using* Ex Taq* DNA polymerase (0.5 U, TaKaRa, Shiga, Japan) in 10 *μ*L of 1x PCR buffer (comprising 2 mM MgCl_2_; TaKaRa) containing 50 ng genomic DNA, 0.1 mM dNTP, and 20 *μ*M of each primer. The PCR conditions were 94°C for 5 min followed by 38 cycles at 94°C for 1 min (or 35 s for primer pair 3/4 at 52°C, 5/7 at 54°C, and Glua3f1/Glua3r2 at 59°C), followed by 72°C for 90 s and a final extension at 72°C for 10 min.

### 2.3. DNA Cloning and Sequencing of LMW-GS Genes

The PCR products were separated using 1.2% agarose gels and the expected fragments were isolated from the gels using an EasyPure Quick Gel Extraction Kit (TransGen, Beijing, China). The isolated PCR products were cloned into the pMD18-T Simple Vector (TaKaRa) and transformed into* E. coli* Competent Cells DH5*α* (TaKaRa). The positive colonies were verified using M13 universal primers, and the selected clones were sequenced by Shanghai Sangon Biological Engineering & Technology and Service Ltd. Each PCR and sequencing analysis was repeated at least three times to avoid technical errors.

### 2.4. Accession Numbers

The LMW-GS gene sequences identified from Jiangdongmen, Daqingmang, Hongjinbaoyin, Dabaimai, Hongmangzi, Hongdougou, and Baimangmai were deposited in GenBank under accession numbers KF020658–KF020665. KF020658 and KF020659 were cloned using* Glu-A3* locus-specific primers from Daqingmang and Hongjinbaoyin, designated as* Glu-A3-1a *and* Glu-A3-1b*, respectively. The sequences KF020660–KF020662 were cloned using* Glu-B3* locus-specific primers from Hongdougou, Dabaimai, and Hongmangzi, designated as* Glu-B3-1a*,* Glu-B3-1b*, and* Glu-B3-1c*, respectively, and KF020663–KF020665 were cloned using* Glu-D3* locus-specific primers from Jiangdongmen, Daqingmang, and Baimangmai, designated as* Glu-D3-1a*,* Glu-D3-1b*, and* Glu-D3-1c*, respectively.

### 2.5. LMW-GS Gene Analysis

Sequence analysis and characterization were performed using DNAMAN software (http://www.lynnon.com/), BLAST tools at NCBI (http://blast.ncbi.nlm.nih.gov/Blast.cgi/), and the PlantCare database (http://bioinformatics.psb.ugent.be/webtools/plantcare/html/). The nomenclature of the LMW-GS genes followed the “Catalogue of Gene Symbols for Wheat” at http://wheat.pw.usda.gov/ggpages/wgc/98//.

## 3. Results and Discussion

### 3.1. Basic Characteristics of the LMW-GS Alleles Identified in This Study

Eight novel LMW-GS alleles with no intron were obtained in this study (see Figure S1 in Supplementary Material available online at http://dx.doi.org/10.1155/2014/371045/). Their sequences were submitted to GenBank with the accession numbers KF020658–KF020665 and designated as* Glu-A3-1a*,* Glu-A3-1b*,* Glu-B3-1a*,* Glu-B3-1b*,* Glu-B3-1c*,* Glu-D3-1a*,* Glu-D3-1b*, and* Glu-D3-1c*, following the nomenclature rules at http://wheat.pw.usda.gov/ggpages/wgc/98//.* Glu-A3-1a* and* Glu-A3-1b *had the same size of 1089 bp and could be translated into 298 amino acids. The nucleotide and deduced amino acid sequences of the other six genes were varied with 1361–1603 bp and 306–351 residues (Table 1). The upstream nucleotide sequences of the cloned genes were searched for in the PlantCare database to analyze the characteristics of the promoter sequences. The eight genes all contained CAAT-boxes and TATA-boxes.* Glu-B3-1a*,* Glu-B3-1b*,* Glu-B3-1c*,* Glu-D3-1a*,* Glu-D3-1b*, and* Glu-D3-1c* contained a Prolamin-box in the upstream sequence (Table 1). All eight genes from landraces in our study belonged to the LMW-m type since their first amino acid at the N-terminal was methionine.

### 3.2. Nucleotide Comparison Analysis of the LMW-GS Alleles within the* Glu-3* Loci

To more accurately compare the differences between LMW-GS genes at the* Glu-3* loci, we used BLAST tools to find the sequences most similar to the eight genes obtained in this study. Nine* Glu-3* genes deposited in GenBank were selected for multiple sequence alignment and included FJ549937, FJ549938, and FJ549946 located at the* Glu-A3* locus; EU369729, EU369730, and DQ630441 at the* Glu-B3* locus; and DQ357054, DQ357055, and EU189094 at the* Glu-D3* locus. Based on the results of these alignments (Table 2), of the eight genes isolated in this study,* Glu-A3-1a*, and* Glu-A3-1b* were found to be new alleles at the* Glu-A3* locus;* Glu-B3-1a*,* Glu-B3-1b*, and* Glu-B3-1c* were new alleles at the* Glu-B3* locus; and* Glu-D3-1a*,* Glu-D3-1b*, and* Glu-D3-1c* were new alleles at the* Glu-D3* locus.

### 3.3. Deduced Proteins Comparison Analysis of the LMW-GS Alleles within the* Glu-3* Loci

The deduced amino acid sequences of the eight genes comprised four structural regions, including a signal peptide of 20 amino acids, a conserved N-terminal region of 13 amino acids, a diverse repetitive domain, and a C-terminal domain, suggesting that the eight genes conformed to the typical molecular characteristics of LMW-GS (Supplementary Material, Figure S2). We compared the alleles within the* Glu-3* loci and found that isoleucine was at the sixth position of the signal peptide in the allele located at* Glu-B3* locus, while valine was at the* Glu-A3* and* Glu-D3* loci (Table 3), which might result from the substitution of ATC for GTC (Supplementary Material, Figure S1). The eleventh position of the N-terminal at the* Glu-A3* and* Glu-D3* loci was arginine, while lysine filled the same position at the* Glu-B3* locus (Table 3), which may be the result of an AGA → AAA transversion (Supplementary Material, Figure S1).

Eight cysteine residues were found in* Glu-A3-1a*,* Glu-A3-1b*,* Glu-B3-1b*,* Glu-B3-1c*,* Glu-D3-1a*,* Glu-D3-1b*, and* Glu-D3-1c*, while* Glu-B3-1a* had nine. By comparing the* Glu-3* loci alleles, the locations of the first and seventh cysteine were found to be varied. As shown in Figure S2 (Supplementary Material) and Table 3, the first cysteine of the* Glu-A3* and* Glu-D3* alleles was located at the fifth position of the N-terminal domain, whereas the first cysteine of the* Glu-B3* alleles was found at the 46th position of the deduced amino acid sequences in the repetitive domain. The seventh cysteine at the* Glu-D3* alleles was located 16 amino acids higher up than that at the* Glu-A3* and* Glu-B3* alleles. In particular, the extra cysteine of allele* Glu-B3-1a* was found at the 321st position of the amino acid sequence.

Usually the first and seventh cysteine residues participate in forming the intermolecular disulfide bond, while the remaining residues participate in the formation of intramolecular disulfide bonds [21]. Disulfide bonds have an important influence on determining the properties and structure of wheat gluten proteins [22]. The first cysteine at the* Glu-B3* allele was in the repetitive domain, however, the first cysteine at* Glu-A3* and* Glu-D3* alleles were in the N-terminal. The positions of the seventh cysteine residue at the* Glu-D3* alleles were also different from the* Glu-A3* and* Glu-B3* alleles. The LMW-GS gene, which had nine cysteine residues, resulted in desirable processing quality [20]; therefore, the new allele of* Glu-B3-1a* may be the desirable LMW-GS gene for improving bread-making quality because the extra cysteine residue could form more disulfide bonds than other alleles during the development of glutenin macropolymer.

Insertions/deletions often appeared within the repetitive domain. The length variation of the LMW-GS genes was mainly due to the numbers of repeat motifs, which ranged from 12 to 25 in the repetitive domain. The repeat motif is shown in Table 4. Two long insertions were present in the* Glu-B3* sequences, that is, 18 amino acid insertions at positions 44–61 and eight amino acid insertions at positions 81–88. The repetitive region was composed of a representative repeat motif P_1-2_FP/SQ_2–6_, of which the number would have an impact on changes in the length of the protein [23]. The length of the diverse repetitive domain of* Glu-B3* alleles was longer than those of* Glu-A3* and* Glu-D3* alleles since more insertions occurred in this domain. Masci et al. [24] indicated that a 42 K LMW-GS would have a good processing quality due to its large repeat domain. The repeat domain through intermolecular interactions between large numbers of glutamine side chains, which are both good hydrogen bond donors and acceptors, may prove useful in increasing the viscosity and elasticity of wheat dough [24]. More repeat motifs in the repetitive domain would lead to a better flour quality [25]. Based on Figure S2 (Supplementary Material), the sequences at the* Glu-B3* locus had 50 more deduced amino acids than those at* Glu-A3* and* Glu-D3* loci. Therefore, genes at the* Glu-B3* locus are more desirable than those at the* Glu-A3* and* Glu-D3* loci.

## 4. Conclusion

This study showed that, of the eight new* Glu-3* alleles added to the LMW-GS gene family,* Glu-B3-1a* had nine cysteine residues and the others had eight. The first cysteine of* Glu-A3* and* Glu-D3* alleles was found in the N-terminal domain, while it was located at the repetitive domain of* Glu-B3 *alleles. The LMW-GS allele of* Glu-B3-1a* can be used as candidate gene for improving bread-making quality because it can form more disulfide bonds.

## Supplementary Material

The supplementary material reported multiple alignments of nucleotide and deduced amino acid sequences of the eight cloned genes, named Figure S1 and Figure S2 respectively.

## Figures and Tables

**Table 1:** Comparison of nucleotide sequences of the eight cloned genes.

LMW-GS genes	Upstream	CDS	Total length	Deduced amino acids	Putative transcription binding sites in the upstream region
*Glu-A3-1a *	195 bp	894 bp	1089 bp	298	CAAT-box, TATA-box, ARE, and MRE
*Glu-A3-1b *	195 bp	894 bp	1089 bp	298	CAAT-box, TATA-box, ARE, and MRE
*Glu-B3-1a *	317 bp	1053 bp	1370 bp	351	CAAT-box, TATA-box, and Prolamin-box
*Glu-B3-1b *	317 bp	1044 bp	1361 bp	348	CAAT-box, TATA-box, and Prolamin-box
*Glu-B3-1c *	317 bp	1050 bp	1367 bp	350	CAAT-box, TATA-box, and Prolamin-box
*Glu-D3-1a *	593 bp	921 bp	1591 bp	307	CAAT-box, TATA-box, and Prolamin-box
*Glu-D3-1b *	605 bp	921 bp	1603 bp	307	CAAT-box, TATA-box, and Prolamin-box
*Glu-D3-1c *	605 bp	912 bp	1594 bp	306	CAAT-box, TATA-box, and Prolamin-box

**Table 2:** Sequence identities of the eight new LMW-GS alleles to the previously reported *Glu-3* genes.

LMW-GS alleles	*Glu-A3* gene accession number			*Glu-B3* gene accession number			*Glu-D3* gene accession number		
	FJ549946	FJ549937	FJ549938	DQ630441	EU369729	EU369730	DQ357054	DQ357055	EU189094
*Glu-A3-1a *	93	92	92	84	84	84	87	87	87
*Glu-A3-1b *	93	92	91	83	83	83	86	86	87
*Glu-B3-1a *	85	85	85	95	84	84	83	83	83
*Glu-B3-1b *	N	85	86	99	85	85	84	84	85
*Glu-B3-1c *	N	85	86	99	85	85	83	83	84
*Glu-D3-1a *	87	88	88	88	85	85	95	95	94
*Glu-D3-1b *	86	87	87	83	82	82	99	99	99
*Glu-D3-1c *	87	87	87	84	82	82	99	99	99

Note: N means that no highly similar sequences were found using BLAST tools.

**Table 3:** Comparison of the deduced amino acid sequences for genes isolated in this study and data from GenBank.

		Sig.	N-ter.		Rep.		C-ter.							
*Glu-A3 *	FJ549937	V	C	R			C	C	C	CC		C		C
	FJ549938	V	C	R			C	C	C	CC		C		C
	FJ549946	V	C	R			C	C	C	CC		C		C
	*Glu-A3-1a *	V	C	R			C	C	C	CC		C		C
	*Glu-A3-1b *	V	C	R			C	C	C	CC		C		C

*Glu-B3 *	EU369729	I		K		C	C	C	C	CC		C		C
	EU369730	I		K		C	C	C	C	CC		C		C
	DQ630441	I		K	C		C	C	C	CC		C		C
	*Glu-B3-1a *	I		K	C		C	C	C	CC		C	C	C
	*Glu-B3-1b *	I		K	C		C	C	C	CC		C		C
	*Glu-B3-1c *	I		K	C		C	C	C	CC		C		C

*Glu-D3 *	DQ357054	V	C	R			C	C	C	CC	C			C
	DQ357055	V	C	R			C	C	C	CC	C			C
	EU189094	V	C	R			C	C	C	CC	C			C
	*Glu-D3-1a *	V	C	R			C	C	C	CC	C			C
	*Glu-D3-1b *	V	C	R			C	C	C	CC	C			C
	*Glu-D3-1c *	V	C	R			C	C	C	CC	C			C

The accession numbers are as follows: FJ549937, FJ549938, and FJ549946 are genes at the *Glu-A3* locus; EU369729, EU369730, and DQ630441 are genes at the *Glu-B3* locus; and DQ357054, DQ357055, and EU189094 are genes at *Glu-D3* locus. I, V, R, K, and C represent isoleucine, valine, arginine, lysine, and cysteine, respectively.

**Table 4:** Repeat motif of repetitive domains in the isolated LMW-GS deduced amino acid sequences.

*Glu-A3-1a *	*Glu-A3-1b *	*Glu-B3-1a *	*Glu-B3-1b *	*Glu-B3-1c *	*Glu-D3-1a *	*Glu-D3-1b *	*Glu-D3-1c *
QQQ	QQQ	QQQ	QQQ	QQQ	QQQ	QQQ	QQQ
PLPPQQ	PLPPQQ	PLPPQQQ	PLPPQQQ	PLPPQQQ	PLPPQQ	PLPPQQ	PLPPQQ
SFSQQ	SFSQQ	PPCSQQQQ	PPCSQQQQ	PPCSQQQQ	TFPQQ	TFPQQ	TFPQQ
PPFSQQQQQ	PPFSQQQQQ	PFPQQQQ	PFPQQQQ	PFPQQQQ	PLFSQQQQQQ	PLFSQQQQQQ	PLFSQQQQQQ
PLPQQ	PLPQQ	PIIILQQ	PIIILQQ	SIIILQQ	LFPQQ	LFPQQ	LFPQQ
PSFSQQQ	PSFSQQQ	SPFSQQQQ	SPFSQQQQ	SPFSQQQQ	PSFSQQQ	PSFSQQQ	PSFSQQQ
PPFSQQQ	PPFSQQQ	PVLPQQQ	PVLPQQQ	PVLPQQQ	PPFWQQQ	PPFWQQQ	PPFWQQQ
PILSQQ	PILSQQ	PVIILQQ	PVIILQQ	PVIILQQ	PILPQQ	PPFSQQQ	PPFSQQQ
PPFSQQQQ	PPFSQQQQ	PPFSQQQQQQQQQQQ	PPFSQQQQ	PPFSQQQQ	PPFSQQQQ	PILPQQ	PILPQQ
PVLPQQ	PVLPQQ	PFTQQQ	PVLPQQ	PVLPQQ	LVLPQQ	PPFSQQQQ	PVLPPQQ
SPFSQQ	SPFSQQQQ	PPFSQQ	PPFSQQQQQQQQ	PPFSQQQQQQQQQQ	PVLPPQQ	LVLPQQ	SPFPQQQQ
LVLPPQQQQQQ	LVLPPQQQQQQ	PPISQQQQQQQQQQQ	PPFSQQQQ	PPFSQQQQ	SPFPQQQQQ	PVLPPQQ	LVQQQ
LVQQQ	LVQQQ	PPISQQQQ	PSSQQ	PSSQQ	LVQQQ	SPFPQQQQQ	
		PPFSQQQQ	PPFPQQ	PPFPQQ		LVQQQ	
		TPFSQQQQ	FPQQQ	FPQQQ			
