# Supplementary material for: Cloning and Characterization of Low-Molecular-Weight Glutenin Subunit Alleles from Chinese Wheat Landraces (Triticum aestivum L.)
Source: ScientificWorldJournal. 2014 Apr 10;2014:371045. doi: 10.1155/2014/371045 (PMC4005046; doi:10.1155/2014/371045)
Supplement: Supplementary file 1 — The supplementary material reported multiple alignments of nucleotide and deduced amino acid sequences of the eight cloned genes, named Figure S1 and Figure S2 respectively. [file 371045.f1.pdf]

Supplementary Data

```
Glu-A3-1a .....................................................    0
Glu-A3-1b .....................................................    0
Glu-B3-1a .....................................................    0
Glu-B3-1b .....................................................    0
Glu-B3-1c .....................................................    0
Glu-D3-1a gtcaccgctgcatcgacatatccatcgactaaacaacggtgacccaattatat   53
Glu-D3-1b gtcaccgctgcatcgacatatccatcgactaaacaacggtgacccaattatat   53
Glu-D3-1c gtcaccgctgcatcgacatatccatcgactaaacaacggtgacccaattatat   53

Glu-A3-1a .......................................GATGCCAACGCCTA   14
Glu-A3-1b .......................................--------------   14
Glu-B3-1a ........................gtaggcaccaactcc-g------t--tg-   29
Glu-B3-1b ........................gtaggcaccaactcc-g------t--tg-   29
Glu-B3-1c ........................gtaggcaccaactcc-g------t--tg-   29
Glu-D3-1a attactagagatctttccttatcagtaggcaccaactcc-g----------g-  106
Glu-D3-1b attactagagatctttccttatcagtaggcaccaactcc-g----------g-  106
Glu-D3-1c attactagagatctttccttatcagtaggcaccaactcc-g--------t-g-  106

Glu-A3-1a ATGGCACACTGGTGGTGGCGCTGTACAACGGCACATTGACACTGCACATCGTT   67
Glu-A3-1b -----------a-----------------------------------------   67
Glu-B3-1a ------t-a-a-----c-a---------------g-------c----------   82
Glu-B3-1b ---a--t-a-a-----c-a---------------g-------c----------   82
Glu-B3-1c ------t-a-a-----c-a---------------g-------c----------   82
Glu-D3-1a ----------a-------tt-----------------a---------------  159
Glu-D3-1b ----------a-------tt-----------------a---------------  159
Glu-D3-1c ----------a-------tt-----------------a---------------  159

Glu-A3-1a GGCAAGGTACGGAGTGCAATGGAAGTCACCGCCTCAAGGTGAGCTATCTGGTG  120
Glu-A3-1b -----------------------------------------------------  120
Glu-B3-1a ----------a-----------------t-a-------t----a---------  135
Glu-B3-1b ----------------------------t-a-------t----a---------  135
Glu-B3-1c ----------------------------t-a-------t----a---------  135
Glu-D3-1a ----g-a-------c-------------t-a-------c--------------  212
Glu-D3-1b ----g-a-------c-------------t-a-------c--------------  212
Glu-D3-1c ----g-a-------c-------------t-a-------c--------------  212

Glu-A3-1a TGGCTGCAAAAAGGTACCCTGTAGTACCTGTTGTTGTTGTTGTTGAGGCTGTT  173
Glu-A3-1b -----------------------------------------------------  173
Glu-B3-1a ---------g--ta--------g---t------------ag-c--ct-accca  188
Glu-B3-1b ---------g--ta--------g---t------------ag-c--ct-accca  188
Glu-B3-1c ---------g--ta--------g---t------------ag-c--ct-accca  188
Glu-D3-1a ----------------------gcc-at-----------ag----........  257
```

```
Glu-D3-1b ----------------------gcc-at-----------ag----........   257
Glu-D3-1c -----------------------cc-at-----------ag----........   257

Glu-A3-1a GACCCAGTTGCTGTTGAGGTTGTTGGAAAGAACATTGTCCGAG......CTGC   220
Glu-A3-1b -------------------------------------------......----   220
Glu-B3-1a attgtt--a-t-----------------------c--------ttgctg----   241
Glu-B3-1b attgtt--a-t-----------------------c--------ttgctg----   241
Glu-B3-1c attgtt--a-t-----------------------c--------ttgctg----   241
Glu-D3-1a ..................................---c-----......t---   270
Glu-D3-1b ..................................---c-----......t---   270
Glu-D3-1c ..................................---c-----......t---   270

Glu-A3-1a TGCTGCGACTGCTGTTGGGATTGGGAGACACCTTGACCTGACTGTTGGGGTTG   273
Glu-A3-1b -----------------------------------------------------   273
Glu-B3-1a ------------------tg---------------g--c--------------   294
Glu-B3-1b ------------------tg---------------g--c--------------   294
Glu-B3-1c ------------------tg---------------g--c--------------   294
Glu-D3-1a -------------------g------a----a---g--ca-------------   323
Glu-D3-1b -------------------g------a----a---g--ca-------------   323
Glu-D3-1c -------------------g------a----a---g--ca-------------   323

Glu-A3-1a TTGCTGCTGAGGTTGGACAAAGCCCTG......TTGTTGTTCTTGCAGGATGA   320
Glu-A3-1b ---------------------------......--------------------   320
Glu-B3-1a ---------------------a-----......--------------------   341
Glu-B3-1b ---------------------a-----......--------------------   341
Glu-B3-1c ---------------------a-----......--------------------   341
Glu-D3-1a c----------a-----tgg-a-----aacctg--------------------   376
Glu-D3-1b c----------a-----tgg-a-----aacctg--------------------   376
Glu-D3-1c c----------a-----tgg-a-----aacctg--------------------   376

Glu-A3-1a TGGAGTAGATGATGGCACGGATTGCTTCATAGCGGGATTGTTCGGGGATTTGC   373
Glu-A3-1b -----------------------------------------------------   373
Glu-B3-1a ---------c---------------c---g-----------------------   394
Glu-B3-1b ---------c---------------c---g-----------------------   394
Glu-B3-1c ---------c---------------c---g-----------------------   394
Glu-D3-1a -------------a-----------c--------------c-g----------   429
Glu-D3-1b -------------a-----------c--------------c-g----------   429
Glu-D3-1c -------------a-----------c--------------c-g----------   429

Glu-A3-1a TGCAATTGCTGGCAACATTGTTGTTGCATCACATGGCAACTGCTCTGCTGCCA   426
Glu-A3-1b --------------g--------------------------------------   426
Glu-B3-1a g---gc--t--------------------------------a-----t-----   447
Glu-B3-1b g---gc--t--------------------------------a-----t-----   447
Glu-B3-1c g---gc--t--------------------------------a-----t-----   447
```

```
Glu-D3-1a g----c------------------------g--------------------a-   482
Glu-D3-1b g----c---------------------------------------------a-   482
Glu-D3-1c g----c---------------------------------------------a-   482

Glu-A3-1a CATCTGTGACCTAGCAAGACGTTGTGGCATTGCTACAGGGCTGCACTGCTGCT   479
Glu-A3-1b -----------------------------------------------------   479
Glu-B3-1a ---t----g---------------c-a------c------a------------   500
Glu-B3-1b ---t--------------------c-a------c---t----a--------t-   500
Glu-B3-1c ---t--------------------c-a------c---t----a--------t-   500
Glu-D3-1a ---t--c--------------------------c-------------------   535
Glu-D3-1b ---t--c--------------c-----------c-------------------   535
Glu-D3-1c ---t--c--------------------------c-------------------   535

Glu-A3-1a GGAGGAATACTTTGCATGGGTTTAACTGCTGCAAAACGGATGGCTGAACAATA   532
Glu-A3-1b -----g------------------g----------------------------   532
Glu-B3-1a ----------c-------------g--------g---a-----a----t--ct   553
Glu-B3-1b -------c--c-------------g------------------t-------c-   553
Glu-B3-1c -------c--c-------------g------------------t-------c-   553
Glu-D3-1a ----------c-------------g--------t--t--------------c-   588
Glu-D3-1b ----------c----------c--g-----------t--------------c-   588
Glu-D3-1c --g------gc-------------g-----------t--------------c-   588

Glu-A3-1a GGGATTTGCTGTTG.......................................   546
Glu-A3-1b --------------.......................................   546
Glu-B3-1a --t-----t-----ctgtgaaaatggtgtttgttgttgctgcgaaaatggtgg   606
Glu-B3-1b --------t-----...tggaaactgt...tggtgttgttgtggaaaaggtgg   600
Glu-B3-1c --------t-----...tggaaactgt...tggtgttgttgtggaaaaggtgg   600
Glu-D3-1a --------t-----.......................................   602
Glu-D3-1b --------t-----.......................................   602
Glu-D3-1c --------t-----.......................................   602

Glu-A3-1a .................................CACAAGTTGTTGTTGTTGT.   565
Glu-A3-1b .................................---------------c---.   565
Glu-B3-1a ctgttgctgctgtgaaattggtggttgttgtgaa-acg--g-----------g   659
Glu-B3-1b ttgttgtgaagatggttgttgctgctgcgaaaatggtg--------------g   653
Glu-B3-1c ttgttgtgaagatggttgttgctgctgcgaaaatggtg--------------g   653
Glu-D3-1a ..................caccagctgttggtgttgtt-----------g-aa   637
Glu-D3-1b ..................caccagctgttggtgttgtt-----------g-aa   637
Glu-D3-1c ..................caccagctgttggtgttgtt------...--g-aa   634

Glu-A3-1a ........TGTTGTGGAGGTAAAACTAGTTGTTGTTGCTGCGAAAATGGTGAT   610
Glu-A3-1b ........------------------------a--------------------   610
Glu-B3-1a taaatggt------t-tt--tgttg-t-------c-----t----t-----g-   712
Glu-B3-1b aaaatggtg-----t-t----g----g------------------------g-   706
```

```
Glu-B3-1c aaaatggtg-----t-t----g----g------------------------g-   706
Glu-D3-1a aaggtgat------------------g-------------t----------g-   690
Glu-D3-1b aaggtgat------------------g-------------t----------g-   690
Glu-D3-1c aaggtgat------------------g-------------t----.-----g-   686

Glu-A3-1a TGTTGC.............G.............................GTAG   621
Glu-A3-1b ------.............-.............................----   621
Glu-B3-1a -----tgaaaacggtggtt-ttgttg...tgtaaatggttgttgttgtt--t-   762
Glu-B3-1b -----ttgttgttgttgtt-ttg......tgaaaatggtggttgttgtg----   753
Glu-B3-1c -----ttgttgttgttgtt-ttgttgttgtgaaaatggtggttgttgtg----   759
Glu-D3-1a ------ggtagaactagtt-ttg.....................ttgct-cga   722
Glu-D3-1b ------ggtagaactagtt-ttg.....................ttgct-cga   722
Glu-D3-1c ------ggtagaactagtt-ttg.....................ttgct-cga   718

Glu-A3-1a AACTGGTTGTTGTTGCTGTGAAAATGGTGGTTGCTGCGATAGAAT...TGGTT   671
Glu-A3-1b ---------------------------------------------...-----   671
Glu-B3-1a ttg-t-------------c--------------t---ag--t---aac-----   815
Glu-B3-1b ------------------c--------------t---ag--t---aac-----   806
Glu-B3-1c ------------------c--------------t---ag--t---aac-----   812
Glu-D3-1a --a----g---...------gt-gaat------t---tgaga--atgg-----   772
Glu-D3-1b --a----g---...------gt-gaat------t---tgaga--atgg-----   772
Glu-D3-1c --a----g---t..------gt-gaat------t---tgaga--atgg-----   769

Glu-A3-1a GTTGCTGCGA...AAATGGTGGTTGTTGCTGCGAAAATGATGGTTGTTGAGGT   721
Glu-A3-1b ----------...----------------------------------------   721
Glu-B3-1a -c--t----gcag--c----t------------------g--a------ca--   868
Glu-B3-1b -c--t----gcag--c----t------------------g--a------ca--   859
Glu-B3-1c -c--t----gcag--c----t------------------g--a------ca--   865
Glu-D3-1a --------c-...---------------------------------------a   822
Glu-D3-1b --------c-...---------------------------------------a   822
Glu-D3-1c --------c-...---------------------------------------a   819

Glu-A3-1a A..ATGGTTG.TTGTTGTTGTT...GTGAAAATGGTGGTTGTTG...TGAAAA   765
Glu-A3-1b -..-------.-----------...-------------------...------   765
Glu-B3-1a -ta--aa---g---------c-...---g-------t-------ctg----c-   918
Glu-B3-1b -ta--aa---g---------c-...---g-------t-------ctg----c-   909
Glu-B3-1c -ta--aa---a---------c-...---g-------t-------ctg----c-   915
Glu-D3-1a -..--a----.-----------gtt--------a----------...--g---   869
Glu-D3-1b -..--a----.-----------gtt--------a----------...--g---   869
Glu-D3-1c -..--a----.--------......--------a----------...-ag---   860

Glu-A3-1a TGAC......TGTTGTGGTGGTAATGGTTGCTGCTGCCATGGTCTCTCCAAAC   812
Glu-A3-1b ----......-------------------------------------------   812
Glu-B3-1a --gtggttgt--------------------t------------t---------   971
```

```
Glu-B3-1b --gtggttgt--------------------t------------t---------  962
Glu-B3-1c --gtggttgt--------------------t------------t---------  968
Glu-D3-1a --t-......-------------------------------------------  916
Glu-D3-1b --t-......-------------------------------------------  916
Glu-D3-1c --t-......-------------------------------------------  907

Glu-A3-1a CAGAGATGCAGCTAGTCTCCATCTGTGCAATGGCACTTGTCGCCACAACGGCG  865
Glu-A3-1b -----------------------------------------------------  865
Glu-B3-1a ---g--c--g------------t----------------c----g---t---a 1024
Glu-B3-1b ---g--c--g------------t---------------------g---t---a 1015
Glu-B3-1c ---g--c--g------------t---------------------g---t---a 1021
Glu-D3-1a ---g------t--------------c-----t------------g--------  969
Glu-D3-1b ---g------t--------------c-----t------------g------t-  969
Glu-D3-1c ---g------t--------------c-----t------------g--------  960

Glu-A3-1a ATGAGGGCAAAGACGAGGAAGATCTTCATGATGGATTGGTGCTAACTACTTTT  918
Glu-A3-1b ---------------------g-------------------------------  918
Glu-B3-1a -g---a-------t-------g--------g----------t------g-a-- 1077
Glu-B3-1b -g---a-------t-------g--------g----------t------g-a-- 1068
Glu-B3-1c -g---a-------t-------g--------g----------t------g-a-- 1074
Glu-D3-1a -g-------------------g--------g----------t------g-g-- 1022
Glu-D3-1b -g-------------------g--------g----------t------g-g-- 1022
Glu-D3-1c -g-------------------g--------g----------t------g-g-- 1013

Glu-A3-1a GCTTGGTTTTGATGCTTGTGCTTGTGATGATCATTATACTTCATGCCTATTTA  971
Glu-A3-1b -----------------------------------------------------  971
Glu-B3-1a --------------a------c----------t-g------------------ 1130
Glu-B3-1b --------------a------c----------t-g------------------ 1121
Glu-B3-1c --------------a------c----------t-g------------------ 1127
Glu-D3-1a --------c-----------------------t-------------------- 1075
Glu-D3-1b --------c-----------------------t-------------------- 1075
Glu-D3-1c --------c-----------------------t-c------------------ 1066

Glu-A3-1a TAGTAGTCCGGGCACCACCTCTTT.CCTTCTTTGCATCAACTTAAATAATTCT 1023
Glu-A3-1b ------------------------.---------------------------- 1023
Glu-B3-1a ------c----------t------.--------c-----g---t-------a- 1182
Glu-B3-1b ------c----------t------.--------c-----g---t-------a- 1173
Glu-B3-1c -----ac---------gt------.--------c-----g---t-------a- 1179
Glu-D3-1a ------c----------t------.--------c-----g---t-------a- 1127
Glu-D3-1b ------c--a--a-----------t--------------g---t--------- 1128
Glu-D3-1c ------c--a--a-----------t--------------g---t--------- 1119

Glu-A3-1a ATTTGGATGCATCAGAAATGATTT..TTTGGTACATTGTGTTAGGTT.GCACA 1073
Glu-A3-1b ------------------------..---------------------.----- 1073
```

```
Glu-B3-1a g--------tt--t------ga--..---------------------.----t  1232
Glu-B3-1b g--------tt--t------ga--..---------------------.----t  1223
Glu-B3-1c g--------tt--t------ga--..---------------------.----t  1229
Glu-D3-1a g--------tt--t------ga--..---------------------.----t  1177
Glu-D3-1b g-----c-c----t-------a--at----t-------g--------t----t  1181
Glu-D3-1c g-----c-c----t-------a--at----t----------------t----t  1172

Glu-A3-1a AGCTACAAAAGCGTAC.....................................  1089
Glu-A3-1b ----------------.....................................  1089
Glu-B3-1a --------gc-tacttttggat....tgatcatcaaagttgctttttagttgt  1281
Glu-B3-1b --------gc-tacttttggat....tgatcatcaaagttgctttttagttgt  1272
Glu-B3-1c --------gc-tacttttggat....tgatcatcaaagttgctttttagttgt  1278
Glu-D3-1a --------gc-tacttttggat....tgatcatcaaagttgctttttagttgt  1226
Glu-D3-1b --------gc-tacttttggatatattgatcatcaaagttgatttttagttgt  1234
Glu-D3-1c --------gc-tacttttggatatattgatcatcaaagttgatttttagttgt  1225

Glu-A3-1a .....................................................  1089
Glu-A3-1b .....................................................  1089
Glu-B3-1a gcatatgattatacacaaagtatagaaatcctaaagtttgctatagatgaatc  1334
Glu-B3-1b gcatatgattatacacaaagtatagaaatcctaaagtttgctatagatgaatc  1325
Glu-B3-1c gcatatgattatacacaaagtatagaaatcctaaagtttgctatagatgaatc  1331
Glu-D3-1a gcatatgattatacacaaagtatagaaatcctaaagtttgctatagatgaatc  1279
Glu-D3-1b gcatacgatcacacacaaagtacagaaaccccgatatttgctatatatgactc  1287
Glu-D3-1c gcatacgatcacacacaaagtacagaaaccccgatatttgctatatatgactc  1278

Glu-A3-1a .....................................................  1089
Glu-A3-1b .....................................................  1089
Glu-B3-1a accttattcactttacatgtcatgcacttctcagga.................  1370
Glu-B3-1b accttattcactttacatgtcatgcacttctcagga.................  1361
Glu-B3-1c accttattcactttacatgtcatgcacttctcagga.................  1367
Glu-D3-1a accttattcactttacatgtcacgcacttatcaggatagtatcacttttgcaa  1332
Glu-D3-1b accttattaactttacatgtcacacacttatcaagatagtatcacttttacaa  1340
Glu-D3-1c accttattaactttacatgtcacacacttatcaagatagtatcacttttacaa  1331

Glu-A3-1a .....................................................  1089
Glu-A3-1b .....................................................  1089
Glu-B3-1a .....................................................  1370
Glu-B3-1b .....................................................  1361
Glu-B3-1c .....................................................  1367
Glu-D3-1a ccgagtttggaaccttgtatgggctgtcagccattaacaagaaagacgaaact  1385
Glu-D3-1b ccgagtttggaaccttgtataggctgtcagccgtaaacaagaaaggcaaaact  1393
Glu-D3-1c ccgagtttggaaccttgtataggctgtcagccgtaaacaagaaaggcaaaact  1384

Glu-A3-1a .....................................................  1089
```

```
Glu-A3-1b ..................................................  1089
Glu-B3-1a ..................................................  1370
Glu-B3-1b ..................................................  1361
Glu-B3-1c ..................................................  1367
Glu-D3-1a tgtgtggaaccaagtc.gcaatcgtgtctcggctattaagatatgctaaagcc  1437
Glu-D3-1b tgtgtatgactaagtttgcaatcatgtgtgggctgttaggatatgctcgatta  1446
Glu-D3-1c tgtgtatgactaagtttgcaatcatgtgtgggctgttaggatatgctcgatta  1437

Glu-A3-1a ..................................................  1089
Glu-A3-1b ..................................................  1089
Glu-B3-1a ..................................................  1370
Glu-B3-1b ..................................................  1361
Glu-B3-1c ..................................................  1367
Glu-D3-1a ttgc.gtgtcgagggt..acggttcttctaggctcggttgcttttcgaaaaac  1487
Glu-D3-1b gtaaagccttgagtgtggagtgttcttctatattcggttgcttttcagaaaac  1499
Glu-D3-1c gtaaagccttgagtgtggagtgttcttctatattcggttgcttttcagaaaac  1490

Glu-A3-1a ..................................................  1089
Glu-A3-1b ..................................................  1089
Glu-B3-1a ..................................................  1370
Glu-B3-1b ..................................................  1361
Glu-B3-1c ..................................................  1367
Glu-D3-1a taactttgtattttcctctaaggtaataagatgaactgacgcctatagagcat  1540
Glu-D3-1b taactttgtactttcctgtaagatgataagatgaactgacgtctatagagcat  1552
Glu-D3-1c taactttgtactttcctgtaagatgataagataaactgacgtctatagagcat  1543

Glu-A3-1a ................................................   1089
Glu-A3-1b ................................................   1089
Glu-B3-1a ................................................   1370
Glu-B3-1b ................................................   1361
Glu-B3-1c ................................................   1367
Glu-D3-1a gacatgactcatcaaatcctctttacatgtaaaggatggcagtttctacaa   1591
Glu-D3-1b gacatgactcatcgaatccgttttacatgtaaaggatggcagtttctacaa   1603
Glu-D3-1c gacatgactcatcgaatccgctttacatgtaaaggatggcagtttctacaa   1594
```

Figure S1: Alignments of the 8 LMW-GS genes nucleotide sequences. Dashes and dots indicate the same sequences and deletions, respectively.

```
Glu-A3-1a MKIFLVFALIAVVATSAIAQMETSCISGLERPWQQQPLPPQQS.........    43
Glu-A3-1b --t----------------------------------------.........    43
Glu-B3-1a --t--i---l-ia-a---------rvp---k-----------qppcsqqqqp    52
Glu-B3-1b --t--i---l-ia-----------rvp---k-----------qppcsqqqqp    52
Glu-B3-1c --t--i---l-ia-----------rvp---k-----------qppcsqqqqp    52
Glu-D3-1a --t------l--a----------r--p---------------t.........    43
Glu-D3-1b --t------lt-a----------r--p---------------t.........    43
Glu-D3-1c --t------l--a----------r--p---------------t.........    43

Glu-A3-1a .........FSQQPPFSQQQQQ.PLPQQ........PSFSQQQ.......PP    70
Glu-A3-1b .........-------------.-----........-------.......--    70
Glu-B3-1a fpqqqqpiiil--s-------p.v----qpviilqq-p-----qqqqqqqq-   103
Glu-B3-1b fpqqqqpiiil--s-------p.v----qpviilqq-p-----qpvlpqq--   103
Glu-B3-1c fpqqqqsiiil--s-------p.v----qpviilqq-p-----qpvlpqq--   103
Glu-D3-1a .........-p---l-------qlf---........-------ppfwqqq--    78
Glu-D3-1b .........-p---l-------qlf---........-------ppfwqqq--    78
Glu-D3-1c .........-l---l------..lf---........-------ppfwqqq--    76

Glu-A3-1a FSQQQPILSQQ.PPFSQQQQPVLPQQSPFSQQQQLVLPPQ......Q.....   110
Glu-A3-1b -----------.--------------------l-------......-.....   110
Glu-B3-1a -t----pf---.--i-----qqqq--q--t---.ppfsq-ppisqq-qppfs   153
Glu-B3-1b -----qqqq-..--------------p-------ppfsq-qqpssq-.ppfp   152
Glu-B3-1c -----qqqq--q--------------p-------ppfsq-qqpssq-.ppfp   154
Glu-D3-1a --------p--.--------l-----p-------p-----qspfpq-.....   124
Glu-D3-1b --------p--.--------l-----p-------p-----qspfpq-.....   124
Glu-D3-1c --------p--.ttifaatttsstattti-----p-----qspfpq-.....   122

Glu-A3-1a QQ..QQLVQQQ.IPIVQPSVLQQLNPCKVFLQQQCSPVAMPQRLARSQMWQQ   159
Glu-A3-1b --..-------.-----------------s----------------------   159
Glu-B3-1a --qqtpfs---q--vih------------------i----s-----p-----   205
Glu-B3-1b --hq-.fp---.--v---------------------h---s-----------   202
Glu-B3-1c --hq-.fp---.--v---------------------h---s-----------   204
Glu-D3-1a --qh-------.--v----im----------------------------l--   175
Glu-D3-1b --qh-------.--v----i----d------------------------l--   175
Glu-D3-1c --.h-------.--v----i--------l-p------------------l--   172

Glu-A3-1a SSCHVMQQQCCQQLQQIPEQSRYEAIRAIIYSIILQEQQ..QGFVQPQQQQP   209
Glu-A3-1b ---------------------------------------..-----------   209
Glu-B3-1a --------------p-------s------v---------..-----------   255
Glu-B3-1b --------------p-------s------v---------..-----------   252
Glu-B3-1c --------------p-------s------v---------..-----------   254
Glu-D3-1a ----a---------p---q--------------------qv--si-s-----   227
Glu-D3-1b --------------p---q--------------------qv--si-s-----   227
Glu-D3-1c --------------p---q--------------------qv--si-s-----   224
```

```
Glu-A3-1a QQSGQGVSQSQQQSQQQ..LGQCSFQQPQ..QQLGQQPQQQQQVLQGTFLQ   257
Glu-A3-1b -----------------..----------..--------------------   257
Glu-B3-1a ---------h-------qq----------ql----------..ip--i---   305
Glu-B3-1b ---------h-------qq----------ql----------..ip--i---   302
Glu-B3-1c ---------h-------qq----------ql----------..ip--i---   304
Glu-D3-1a --l--c---p------...............----------..la------   261
Glu-D3-1b --l--c---p------...............----------..la------   261
Glu-D3-1c --l--c---p------...............----------..lv------   258

Glu-A3-1a PHQIAHLEAVTSIALRTLPTMCSVNVPLYSATTSVPLGVGI.....        298
Glu-A3-1b --------------------------------i--------.....        298
Glu-B3-1a ----sq--vm-----c------g-------s--im-fsi-tgvgay        351
Glu-B3-1b ----sq--vm------------g-------s--imsfsi-tgvgay        348
Glu-B3-1c ----sq--vm------------g-------s--im-fsi-tgvgay        350
Glu-D3-1a -----q--vm------i------------rt-----f---tgvgay        307
Glu-D3-1b -----q--vm------i------------rt-----f---tgvgay        307
Glu-D3-1c -----q--vm------i------------rt-----fd--tgvgay        304
```

Figure S2: Multiple alignments of the deduced amino acid sequences of eight cloned genes in the study. The same sequences and deletions indicated by dashes and dots, respectively.
